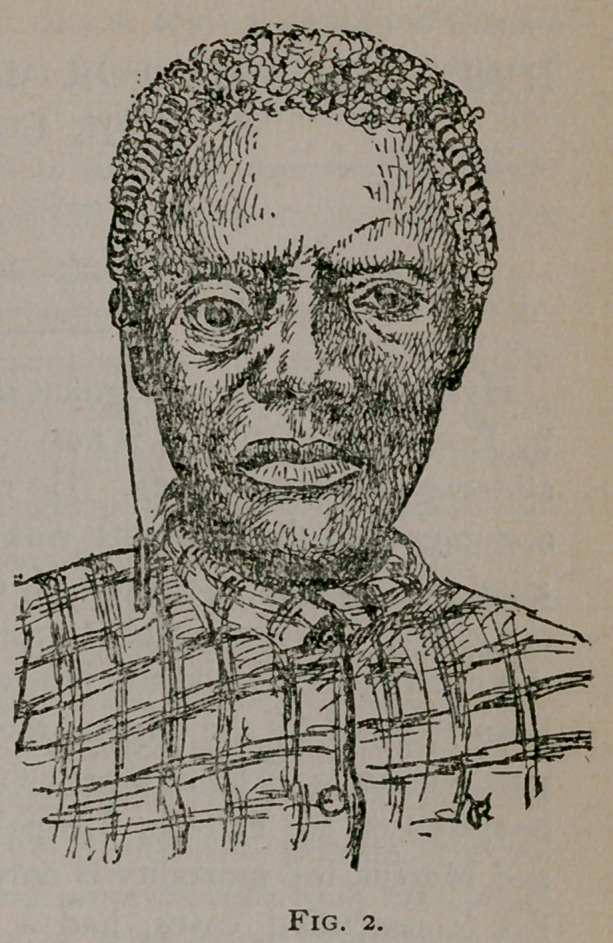# Myxomata of the Orbit with Colloid Degeneration

**Published:** 1888-11

**Authors:** W. L. Bullard

**Affiliations:** Columbus, Ga.


### MYXOMATA OF THE ORBIT WITH COLLOID DEGENERATION.

BY W. L. BULLARD, M. D., COLUMBUS, GA.

These cuts are from photographs of S. M., female, æt. 69, whose case I reported in the December number of this journal of 1887 (*quod vide*). It is now about one year since I operated, and she being in the city a few days ago called to see me, and there being such a favorable change in her condition, both physically and cosmetically, so to speak, that I again had her photographed.

FIG. 1.

FIG. 2.

The cut No. 2 shows her condition now, which is quite an improvement. The opaque cornea, since replaced into the socket, has become almost transparent, and while she has no control over the eye (the recti muscles having been absorbed from colloid degeneration), yet it is quite deceptive, and the old woman is unquestionably elated over her present condition. For the benefit of those who are not interested specially in eye surgery or orbital tumors, allow me to say parenthetically that when we consider the rarity of this kind of growth in this situation, together with its successful termination after surgical interference, it is indeed an interesting and unique case.

## Figures and Tables

**Fig. 1. f1:**
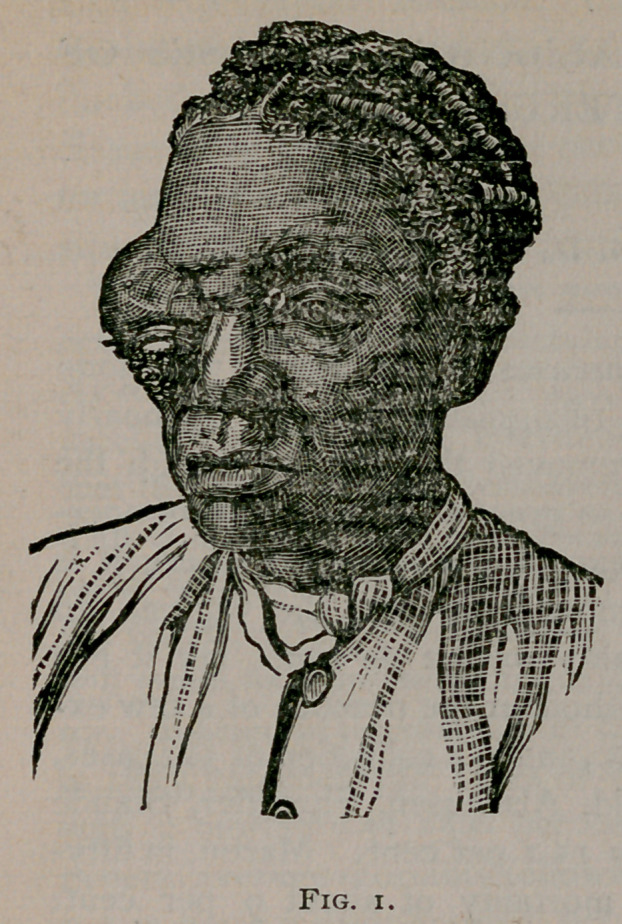


**Fig. 2. f2:**